# The Effect of *Curcuma phaeocaulis* Valeton (Zingiberaceae) Extract on Prion Propagation in Cell-Based and Animal Models

**DOI:** 10.3390/ijms24010182

**Published:** 2022-12-22

**Authors:** Sungeun Lee, Hakmin Lee, Jaehyeon Kim, Ji Hoon Kim, Eun Mei Gao, Yoonjeong Lee, Miryeong Yoo, Trang H. T. Trinh, Jieun Kim, Chul Young Kim, Chongsuk Ryou

**Affiliations:** Department of Pharmacy, College of Pharmacy and Institute of Pharmaceutical Science & Technology, Hanyang University, 55 Hanyangdaehak-ro, Sangnok-gu, Ansan 15588, Gyeonggi-do, Republic of Korea

**Keywords:** prions, inhibition, *Curcuma phaeocaulis* Valeton, natural products

## Abstract

Prion diseases are neurodegenerative disorders in humans and animals for which no therapies are currently available. Here, we report that *Curcuma phaeocaulis* Valeton (Zingiberaceae) (*CpV*) extract was partly effective in decreasing prion aggregation and propagation in both in vitro and in vivo models. *CpV* extract inhibited self-aggregation of recombinant prion protein (PrP) in a test tube assay and decreased the accumulation of scrapie PrP (PrP^Sc^) in ScN2a cells, a cultured neuroblastoma cell line with chronic prion infection, in a concentration-dependent manner. *CpV* extract also modified the course of the disease in mice inoculated with mouse-adapted scrapie prions, completely preventing the onset of prion disease in three of eight mice. Biochemical and neuropathological analyses revealed a statistically significant reduction in PrP^Sc^ accumulation, spongiosis, astrogliosis, and microglia activation in the brains of mice that avoided disease onset. Furthermore, PrP^Sc^ accumulation in the spleen of mice was also reduced. *CpV* extract precluded prion infection in cultured cells as demonstrated by the modified standard scrapie cell assay. This study suggests that *CpV* extract could contribute to investigating the modulation of prion propagation.

## 1. Introduction

Prion diseases, also known as transmissible spongiform encephalopathies, are a group of transmissible diseases caused by non-nucleic acid proteinaceous pathogens called prions [1,2]. These diseases affect various mammalian species, including humans. The representative prion diseases are scrapie in sheep, bovine spongiform encephalopathy in cattle and Creutzfeldt–Jakob disease (CJD) in humans [3,4,5]. The disease-causing agents, prions, are composed of misfolded isoforms of prion protein (PrP), referred to as scrapie PrP (PrP^Sc^), which is formed from cellular PrP (PrP^C^) through conformational conversion [2]. PrP^Sc^ forms a variety of aggregates, such as oligomers and amyloids [6,7]. Neuropathologically, PrP^Sc^ aggregate deposits are often found as amyloid plaques in the lesions of affected brains [8,9]. PrP^Sc^ aggregates induce the death of neurons, leading to vacuolation and eventually spongiform degeneration, and they promote the activation of glial cells (known as astrogliosis) in the brain as the disease progresses [10].

*Curcuma phaeocaulis* Valeton (Zingiberaceae) (*CpV*) has been used as a traditional medicine for the treatment of blood stasis syndrome due to stagnant blood, such as psychataxia, dysmenorrhea and arthralgia in East Asian regions [11]. Recent phytochemical investigations of this plant showed that sesquiterpenoid, one of the main constituents of *CpV* [12], exhibits anti-inflammatory [13], anti-tumor [14], and anti-platelet aggregation activities [15]. However, the effect of *CpV* on brain disorders has not been determined, although curcumin abundantly found in *Curcuma longa* was found effective in inhibiting prion accumulation in vitro [16].

Although prion diseases are known to be unexceptionally fatal [17,18,19], no effective therapy is available. To date, the development of treatments for prion disease has been limited to laboratory investigation due to the inefficacy and toxicity of the agents employed in human trials [20,21]. For example, quinacrine effectively decreased the level of PrP^Sc^ in cultured neuroblastoma cells with chronic prion infection [22,23,24] but was not efficacious in a mouse model of prion disease and CJD patients, resulting in liver failure [25,26,27]. Although polypentosan sulfate demonstrated a certain level of efficacy in an experimental animal model, in a human trial, it was also determined to be inappropriate as a treatment option for diseases because the effect was limited to isolated cases [27,28,29,30,31]. Currently, the unmet need to develop an innovative treatment option for prion diseases is still not resolved.

In this study, *CpV* extract was investigated using an in vitro assay that reflects the conversion and aggregation of PrP molecules. Moreover, the anti-prion activity of *CpV* extract was determined in cultured cells and in a mouse model of prion disease. The effect of *CpV* extract was confirmed at the biochemical and neuropathological levels.

## 2. Results

### 2.1. CpV Extract Inhibited Aggregation of Unfolded/Misfolded rPrP In Vitro

**Figure 1:**
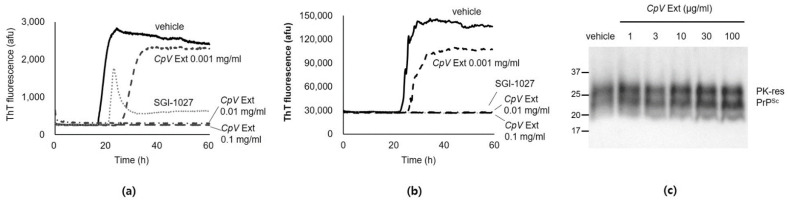
Inhibition of unfolded/misfolded rPrP aggregation by *CpV* extract. (**a**) Formation of guanidine hydrochloride-unfolded rPrP aggregates was monitored in the PAFA reactions with 0.001–0.1 mg/mL *CpV* extract (Ext). The PAFA with SGI-1027 (100 μM) was used as a positive control. afu, arbitrary fluorescence unit. (**b**) Aggregation of misfolded rPrP converted from normal conformers through PrP^Sc^ seeding was monitored using RT-QuIC assay with 0.001–0.1 mg/mL *CpV* extract. SGI-1027 was used as a positive control compound. (**c**) Western blotting of PK-resistant PrP^Sc^ in SBH incubated with 1–100 μg/mL *CpV* extract.

Initially, PrP amyloid formation assay (PAFA) was performed to address the effect of CpV extract on recombinant (r) PrP self-aggregation. CpV extract interfered with self-aggregation of chemically unfolded rPrP in a concentration-dependent manner, exhibiting complete inhibition when > 0.01 mg/mL CpV extract was present in the PAFA reactions (Figure 1a). This was more efficacious than the inhibition by SGI-1027, the effect of which was previously reported [32]. Next, real-time quaking-induced conversion (RT-QuIC) was performed to investigate if CpV extract could inhibit rPrP conversion through PrP^Sc^ seeding. Just as the control compound SGI-1027, CpV extract > 0.01 mg/mL completely blocked aggregation of misfolded rPrP that was generated by conversion of rPrP through seeded PrP^Sc^ (Figure 1b). This effect was concentration-dependent. To investigate the effect of CpV extract on the stability of PrP^Sc^, the level of PrP^Sc^ in brain homogenate of mouse-adapted RML scrapie prion-ill mice (called RML-sick brain homogenate (SBH) hereafter) was determined using western blotting after overnight incubation with CpV extract. The level of proteinase K (PK)-resistant PrP^Sc^ was unchanged, suggesting that CpV extract could not destabilize PrP^Sc^ to be PK-digestible (Figure 1c).

### 2.2. CpV Extract Decreased the Level of PrP^Sc^ in ScN2a Cells

**Figure 2:**
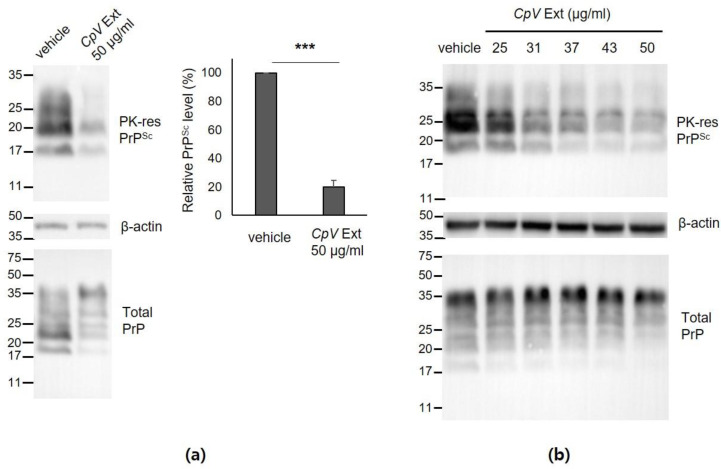
Anti-prion activity of *CpV* extract in ScN2a cells. (**a**) A representative western blot and densitometry analysis of multiple western blots (n = 4), demonstrating a reduction in PK-resistant (PK-res) PrP^Sc^ level in ScN2a cells by *CpV* extract. ***, *p* = 0.000007. (**b**) The *CpV* extract concentration-responsive decrease in PK-resistant PrP^Sc^ in ScN2a cells. β-actin was used a loading control. Total PrP was detected from ScN2a cell lysate without PK digestion.

A cell-based PrP^Sc^ assay was performed in ScN2a cells, an immortal neuroblastoma cell line with persistent prion infection, to evaluate the anti-prion efficacy of CpV extract [33,34]. Prior to PrP^Sc^ assay, cytotoxicity of CpV extract was determined to estimate the concentrations that are not harmful to the corresponding neuronal cell line. The MTT assay revealed that CpV extract below 50 μg/mL was not cytotoxic (Figure S1). In the PrP^Sc^ assay, the level of PK-resistant PrP^Sc^ in ScN2a cells incubated with 50 μg/mL CpV extract for 4 days was significantly reduced below ~20% of that found in control incubated with vehicle (Figure 2a). This effect occurred in a concentration-dependent manner (Figure 2b), although the highest non-cytotoxic concentration of CpV extract was not able to completely remove PrP^Sc^ in ScN2a cells under the experimental conditions used in this study. The total PrP blot showed that CpV extract was not able to change the overall level of PrP molecules, except for PrP^Sc^ (Figure 2a,b).

### 2.3. CpV Extract Was Tolerable in Mice

To determine whether administration of *CpV* extract causes harmful side effects, the activity of liver enzymes was examined in normal, healthy mice that received *CpV* extract prior to in vivo efficacy tests. Measurements of aspartate aminotransferase (AST) and alanine aminotransferase (ALT) activity in the serum revealed that enzyme activity remained low within the normal range for both (Figure S2a). The enzyme activity was not concentration-dependent. Similarly, to determine whether *CpV* extract was tolerated in prion-infected mice that received *CpV* extract during efficacy tests, the level of liver toxicity was evaluated by measuring AST, ALT, alkaline phospha-tase (ALP) and albumin (ALB) in the serum collected from mice of different groups immediately before euthanasia. Serum biochemistry tests revealed that the liver toxicity marker levels in mice of *CpV* extract-administered groups were not significantly different from those in mice of vehicle-administered groups (Figure S2b). Although the levels were elevated compared to those found in uninfected, healthy counterparts presumably due to prion infection, they still fell within the normal range. This suggest that *CpV* extract administered to mice did not induce severe in vivo toxicity in this study.

### 2.4. CpV Extract Modified the Course of Prion Disease

**Figure 3:**
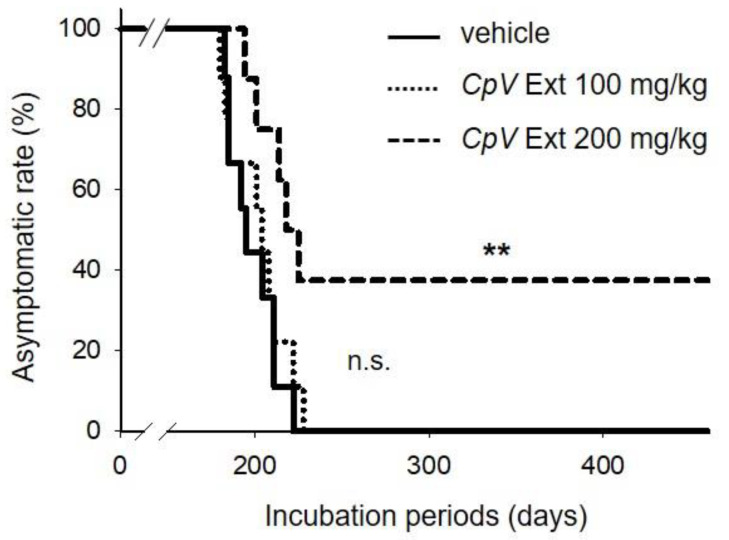
Suppression of prion disease in vivo by *CpV* extract. The disease onset curve of the prion-infected mouse groups (n = 9/group) that received 0, 100, or 200 mg/kg *CpV* extract twice a week for four weeks. **, *p* = 0.009 (vehicle group versus *CpV* Ext 200 mg/kg group); n.s., statistically not significant (vehicle group versus *CpV* Ext 100 mg/kg group).

Next, the anti-prion efficacy of CpV extract administered orally three times a week for four weeks beginning from the day after prion inoculation was evaluated in mice intraperitoneally inoculated with RML-SBH. Administration of 100 mg/kg CpV extract resulted in a week’s prolongation of the incubation period compared to that of the control group that received vehicle only, although not statistically significant (Table 1, Figure 3). On the other hand, in the group that received 200 mg/kg CpV extract, three out of eight mice remained healthy until the end of the experiment at 452 dpi, indicating the prevention of disease onset (Table 1, Figure 3). The remaining five out of eight mice exhibited signs of prion disease. Although their average incubation period was prolonged over ten days compared to that of the control group, this prolongation was not statistically significant. A comparison between whole groups using the log rank test showed that prolongation of incubation period of mice in the group that received 200 mg/kg CpV extract was statistically significant (Table 1, Figure 3, p = 0.009).

### 2.5. CpV Extract Reduced PrP^Sc^ Accumulation in Mouse Brains

**Figure 4:**
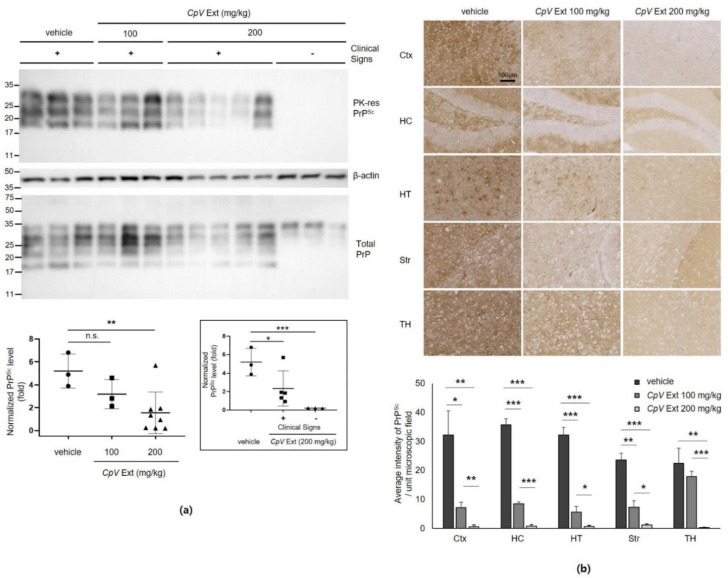
Decrease in PrP^Sc^ in the brains of prion-infected mice that received *CpV* extract. (**a**) The level of PK-resistant (PK-res) PrP^Sc^ in the mouse brains of each group collected at the end point of individual mouse. β-actin was used as loading control. Total PrP was detected from brain homogenate without PK digestion. The western blot of PK-res PrP^Sc^ was quantified with densitometry. The PrP^Sc^ level normalized with the β-actin level was plotted and compared between control and each treated group. **, *p* = 0.008; n.s., statistically not significant. The subgroups with (+) or without (−) clinical signs within 200 mg/kg group were divided and compared individually to the control group (inset). *, *p* = 0.046; ***, *p* = 3.799 × 10^−10^. (**b**) PrP^Sc^ immunohistochemistry and quantification of PrP^Sc^ accumulation. A unit microscopic field is 0.245 mm^2^. *, *p* < 0.05; **, *p* < 0.01; ***, *p* < 0.001. Ctx, cerebral cortex; HC, hippocampus; HT, hypothalamus; Str, striatum; TH, thalamus.

Accumulation of PrP^Sc^ in the brain of mice was assessed using western blotting. The level of PrP^Sc^ in the brain of mice that received 100 mg/kg CpV extract marginally decreased compared to that shown in the mouse brain of the control group (Figure 4a). However, this decrease was not statistically significant. On the other hand, although it varied, the level of PrP^Sc^ in the brain of mice that received 200 mg/kg CpV extract was significantly reduced (Figure 4a). In this group, the PrP^Sc^ level in the brain of mice ill with prion disease decreased below the level detected in the mouse brain of the control group. Most interestingly, no PrP^Sc^ was detected by western blotting in the brain of mice that received 200 mg/kg CpV extract and survived until the end of the experiment. The PrP with molecular mass of 33–35 kD, which mostly represents PrP^C^, was commonly detected in all samples of each group in the total PrP blot, suggesting that CpV extract did not affect PrP^C^ expression in mouse brains (Figure 4a). PrP^Sc^ immunohistochemistry of mouse brain sections from each group that received CpV extract also revealed that the PrP^Sc^ accumulation decreased in various regions of brain in a concentration-dependent manner (Figure 4b).

### 2.6. CpV Extract Reduced Neuropathological Deteriorations

**Figure 5:**
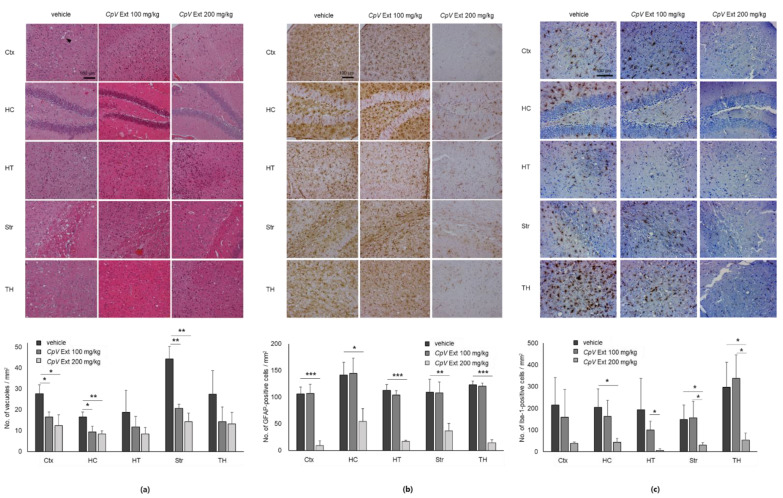
Improved neuropathology of the different brain regions of prion-infected mice that received *CpV* extract. (**a**) H&E staining and quantification of vacuolation. The arrowhead indicates a vacuole. (**b**) GFAP immunohistochemistry and quantification of astrogliosis. (**c**) Iba-1 immunohistochemistry and quantification of microglia activation. *, *p* < 0.05; **, *p* < 0.01; ***, *p* < 0.001. Ctx, cerebral cortex; HC, hippocampus; HT, hypothalamus; Str, striatum; TH, thalamus.

To investigate the effect of *CpV* extract on the neurodegeneration that occurs in the course of prion disease, vacuolation, gliosis, and microglia activation were reviewed in the brain sections of prion-infected mice that received no or 100–200 mg/kg *CpV* extract. In particular, the altered pathologies in the brain sections of mice that received 200 mg/kg *CpV* extract and avoided disease onset were examined. H&E staining demonstrated that the vacuolation found in different brain regions of mice in the control group became gradually lower in those of mice that were given *CpV* extract (Figure 5a). The lesions profiled by counting the number of vacuoles in both groups of 100 and 200 mg/kg administration showed that *CpV* extract decreased spongiform degeneration to a statistically significant level in the cortex, hippocampus, and striatum, but not in the hypothalamus and thalamus. Specifically, the reduction in vacuolation was ~40–55% in the cortex, 43–49% in the hippocampus, and 54–68% in striatum, depending on the dosage (Figure 5a). Glial fibrillary acidic protein (GFAP) immunohistochemistry was performed to examine astrogliosis in the mouse brains. Astrocyte activation progressed dramatically less in every region of the brain of all three mice that received 200 mg/kg *CpV* extract, having thus avoided disease onset, compared to that found in the vehicle- and 100 mg/kg *CpV* extract-administered mice (Figure 5b). Quantification of those GFAP-positive astrocytes demonstrated ~62–91% reductions in astrogliosis that depended on different regions of mouse brains in 200 mg/kg *CpV* extract-administered group. Immunohistochemistry of ionized calcium-binding adapter molecule 1 (Iba-1), a marker of microglia activation, demonstrated reduced Iba-1 expression in every region of the brain of mice that received *CpV* extract (Figure 5c). In particular, quantification of nucleated cells with Iba-1 expression in all three mice that received 200 mg/kg and avoided disease onset revealed statistically significant ~79–82% decreases in the hippocampus, striatum, and thalamus.

### 2.7. CpV Extract Decreased PrP^Sc^ Accumulation but Did Not Affect Follicular Dendritic Cells in Mouse Spleens

**Figure 6:**
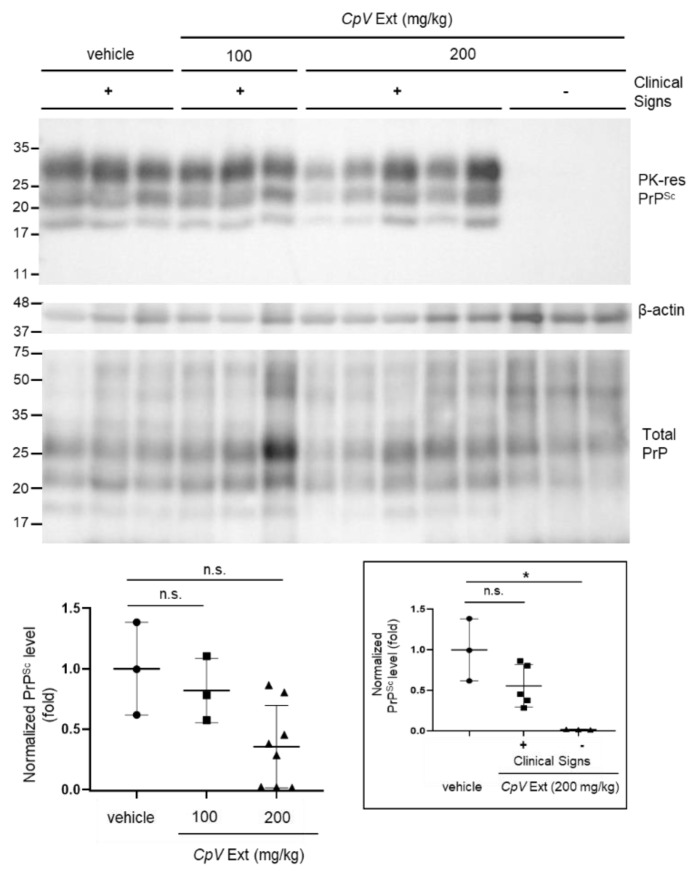
Decrease in PrP^Sc^ in the spleens of prion-infected mice that received *CpV* extract. The level of PK-resistant (PK-res) PrP^Sc^ in the mouse spleens of each group collected at the end point of individual mouse. β-actin was used a loading control. Total PrP was detected from spleen homogenate without PK digestion. The western blot of PK-res PrP^Sc^ was quantified by densitometry. The PrP^Sc^ level normalized with the β-actin level was plotted and compared between control and each treated group. The subgroups with (+) or without (−) clinical signs within 200 mg/kg group were divided and compared individually with the control group (inset). n.s., statistically not significant; *, *p* = 0.011.

To address whether reduction in PrP*^Sc^* in mouse brain correlated with the prevention of PrP*^Sc^* accumulation in the spleen, the level of PK-resistant PrP*^Sc^* in the spleen of mice that received *CpV* extract was assessed using western blotting. The PrP*^Sc^* level of 100 mg/kg *CpV* extract-received group remained similar to that of vehicle-received control group (Figure 6). The splenic PrP*^Sc^* level of mice that received 200 mg/kg *CpV* extract but showed clinical signs of prion disease varied among individuals. Some mice, however, individually demonstrated an obvious decrease in splenic PrP^Sc^ level, although not statistically significant as a group. In contrast, PrP^Sc^ was not accumulated in the spleen of mice that received 200 mg/kg *CpV* extract and showed no clinical signs of prion disease. This indicates that that efficacy of *CpV* extract could be mediated by its effect on the function of certain cells in the spleen. We investigated whether *CpV* extract alters the activation of follicular dendritic cells (FDCs), crucially involved in PrP^Sc^ transmission, by determining the level of FDC-M1, a specific marker of FDCs, in the spleen of mouse that received *CpV* extract. FDC-M1 expression stimulated by prion infection was unaltered by administration of *CpV* extract (Figure S3). This implies that splenic FDCs were not affected.

### 2.8. CpV Extract Precluded Prion Infection In Vitro

**Figure 7:**
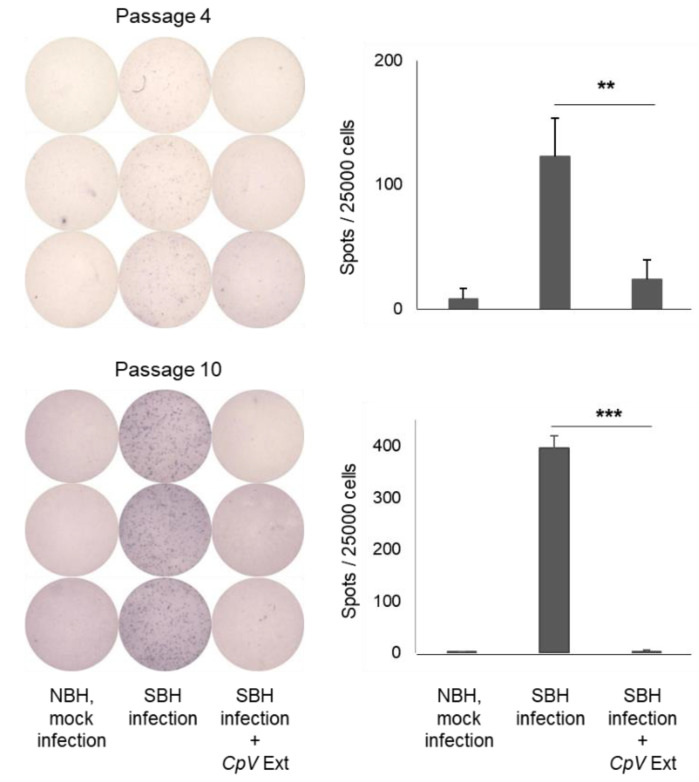
Reduction in prion infectivity by *CpV* extract. SSCA at passages 4 and 10 post-prion infection was used for PrP^Sc^-positive spot counts. **, *p* = 0.008; ***, *p* = 0.00001. NBH, normal brain homogenate (prepared from mice with no prion infection); SBH, sick brain homogenate (prepared from ill mice at the terminal stage of disease due to prion infection).

To clarify the preventive effect of CpV extract to prion infection, cell-based prion infection was performed in the presence of 50 μg/mL CpV extract. Incubation of N2a cells with RML-SBH for 24 h followed by culture of 4 passages in prion-free media resulted in the emergence of PrP^Sc^-positive cells (Figure 7). However, the number of PrP^Sc^-positive cells substantially decreased when prion infection was carried out in the presence of CpV extract. Such an effect became more obvious when prion-infected N2a cells at passage 4 grew an additional 6 passages in the prion-free media, demonstrating an almost complete inhibition of prion infection and propagation within the cell population.

## 3. Discussion

To date, no treatment option for prion disease is available. In previous studies, various agents, mainly chemical compounds, were evaluated in several model systems for prion disease [23,27,35,36]. A small number of compounds among them were further investigated for human patients [21,26,30]. More recently, treatments based on antibody therapy [37], antisense oligonucleotides therapy [38], and gene therapy [39] have been suggested. However, the clinical significance of these approaches has not been confirmed in clinical trials, leaving cures yet to be developed [37]. In this study, we evaluated the efficacy of extract from the natural source in the in vitro and in vivo models of prion disease. This study represents a peculiar attempt to investigate the option for the control of prion propagation by applying the agent of a distinctive source that is different from chemicals and biologicals. Natural product therapeutics have been proven to be safe and effective for a long time, which means they are advantageous in developing a new drug, although the safety and efficacy verification is essential in the context of contemporary medicine [40,41,42]. The FDA has approved the use of tea tree extracts Veregen (sinecatechins onement) [43] and Fulyzaq (Crofelemer) [44] for the treatment of genital warts and diarrhea, respectively.

This study is not the first investigation into using natural extract to control prion diseases. However, this study does demonstrate the most potent efficacy to completely prevent the onset of prion disease in some mice, which has not been the case for other attempts. Previously, some natural extract and compounds, including polyphenols, flavonoids and stilbenoids, were found to exert a prion inhibitory effect in vitro, while they did not reproduce the efficacy in vivo, either failing to increase the incubation time or failing to prevent the disease [45,46,47,48,49]. Interestingly, curcumin is a polyphenol natural product that prevents prion protein fibrillization in vitro, just as it does for amyloid β peptide [50,51], and inhibits PrP^Sc^ accumulation in prion-infected cell lines [16]. Curcumin is isolated from the *Curcuma longa* species, a member of the ginger family, Zingiberaceae. This inspired us to investigate *CpV*, which belongs to the same family, even though the curcumin content in *CpV* is extremely small [52].

Indeed, *CpV* extract tested in PAFA showed effective inhibition of chemically unfolded rPrP self-aggregation. Similarly, *CpV* extract also resulted in the inhibition of aggregation of misfolded rPrP generated by PrP^Sc^ seed-mediated conversion in RT-QuIC, Furthermore, our ScN2a cell-based assay results clearly demonstrate that *CpV* extract suppresses PK-resistant PrP^Sc^ propagation, while a complete elimination of PrP^Sc^ from the cells was impossible within the non-cytotoxic concentration range. Nonetheless, a partial reduction in PrP^Sc^ levels within the non-cytotoxic concentration range suggest that suppression of PrP^Sc^ in ScN2a cells is not directly associated with the decrease in cell numbers but involves a biological mechanism that occurs in the cells. Thus, it provides a solid foundation to evaluate the efficacy of *CpV* extract in an animal model of prion diseases.

Based on our in vitro study results, it is possible to hypothesize that *CpV* extract exerts anti-prion activity by blocking PrP conversion through interaction with abnormal PrP conformers, including PrP^Sc^. PAFA results support the interaction of *CpV* extract with unfolded PrP conformers, leading to inefficient aggregation. RT-QuIC results indicate that interaction of *CpV* extract with PrP^Sc^ seeds hinders conversion of normal PrP conformers to misfolded isoforms, also leading to inefficient aggregation. Nonetheless, *CpV* extract does not appear to affect the stability of PrP^Sc^, as shown in destabilization assay. On the other hand, the effect of *CpV* extract on PrP^C^ remains elusive with the currently available data in this study. In the total PrP blot, the brains of mice with no clinical signs in the 200 mg/kg group showed that the level of lower molecular weight PrP bands (25, 28 kDa) was significantly reduced. Because the lower molecular weight PrP species are accumulated when most of PrP is resistant to degradation, no such bands in the samples could be the result of reduced PrP^C^ expression by the effect of *CpV* extract. Investigation into whether *CpV* extract affects PrP^C^ expression in healthy cells and animals remains to be conducted.

Our in vivo efficacy test results suggest that administration of *CpV* extract in prion-infected mice modified the course of the disease, especially in the group that received 200 mg/kg *CpV* extract. In particular, three of eight mice in the group did not develop the clinical onset of disease, suggesting a possibility of effective pharmacological intervention. In addition, because administration of *CpV* extract with the dosage used in this study was tolerated in mice, the regimen used in this study is suitable for modifying the course of prion disease without causing major side effects. Analyses of the brain from mice with no disease onset support the clinical efficacy of *CpV* extract. Administration of *CpV* extract resulted in almost no accumulation of PrP^Sc^ in the brain, indicating a complete alteration of the prion-associated biochemical event in the course of the disease. Furthermore, the decreases in vacuolation, gliosis, and microglia activation found in mouse brains indicate that administration of *CpV* extract effectively prevented brain damage that occurs in prion disease. Although the effect was not ubiquitous within the group, a complete prevention of prion propagation and corresponding biochemical and neuropathological improvement have not been the case in most in vivo efficacy tests conducted previously [33,53,54], in which a short prolongation of incubation periods were appreciated efficacious. Thus, this study represents a rare example of effective options to modify the course of the disease.

A pitfall associated with in vivo efficacy test can be human errors during the prion infection procedure. To avoid such errors in prion infection in this study, prion- and mock inocula were prepared and injected separately, group by group, by a pair of researchers who practiced independent double checking. Nonetheless, those that remained healthy described above could be the subject of an argument on whether they were actually prion-inoculated. Because prions were intraperitoneally inoculated, retrospective confirmation of prion inoculation by finding the needle track in the brain section was impossible. Thus, the efficaciousness of *CpV* extract to reduce prion infection was reiterated using the in vitro prion infection at the cellular level. The results of the in vitro prion infection confirmed the reduction in prion infectivity using *CpV* extract. Thus, these data dispel the concern that asymptomatic mice with no PrP^Sc^ accumulation and improvement of neuropathology in the brain were the outcomes of human error in the prion infection step during the animal experiment.

In this study, *CpV* extract was administered to mice just after intraperitoneal prion infection. Thus, it is likely that *CpV* extract exerts its effect mainly in the peripheral organs, such as the spleen and lymph nodes, before prion neuroinvasion, resembling successful prophylactics. In support of this hypothesis, PrP^Sc^ accumulation in the spleen and brain of individual mice that received *CpV* extract was almost identical in the current study. These results suggest that *CpV* extract administered for four weeks during the early period of the disease’s time course effectively suppressed the propagation of PrP^Sc^ in the spleen, thus limiting prion spread to the central nervous system. It is speculated that when administration of *CpV* extract was withdrawn, prion suppression in the spleen became ineffective and prion neuroinvasion began in a delayed manner, resulting in the prolongation of the incubation period and, in the best case, no prion propagation in the brain. Therefore, administration of *CpV* extract throughout the entire incubation period would result in improved efficacy in vivo.

Nonetheless, as a preliminary approach, the study described in this communication requires further validation to move on to the next stage in the future. First, the effect of *CpV* extract needs to be tested in a model established with intracerebral prion infection and *CpV* extract administration ideally starting after the clinical signs occur. Second, identification of the active compound within *CpV* extract should be pursued to validate the efficacy and the mode of action of *CpV* extract.

## 4. Materials and Methods

### 4.1. Preparation of CpV Extracts

The rhizomes of *CpV* (Figure S4a) were purchased from the Kyungdong Oriental herbal market, Seoul, Republic of Korea, in July 2018. A voucher specimen (HYUP-Cpv-001) was deposited in the Herbarium of the College of Pharmacy, Hanyang University. *CpV* (600 g) was extracted with methanol through reflux for 2 h. Extraction was repeated three times and was combined. The filtrate was evaporated under reduced pressure using a rotary evaporator to obtain 31.1 g of crude extract. *CpV* extract prepared and employed in this study demonstrated the HPLC chromatogram with a characteristic content profile reported elsewhere (Figure S4b) [12]. Curcumin abundantly included in *Curcuma longa* extract was a trace component in *CpV* extract. The extract was saved at −80 °C until used and was dissolved in dimethyl sulfoxide (DMSO) when applied to assays.

### 4.2. PAFA

PAFA, an in vitro prion aggregation assay that monitors the rPrP self-aggregation, was performed as described previously [55]. In this study, *CpV* extract (0.001–0.1 mg/mL) was mixed in 200 μL of PAFA reaction buffer [0.2 M guanidine hydrochloride, 10 μM thioflavin T (ThT) in PBS (pH 7.2)] with guanidine hydrochloride-denatured 50 μM mouse rPrP substrate. The reaction mixture was incubated at 42 °C for 60 h with continuous shaking at 700 rpm. The PrP aggregates were detected by the reading of ThT fluorescence intensity every 15 min using an Infinite M200Pro multimode reader (Tecan, Männedorf, Switzerland).

### 4.3. RT-QuIC

RT-QuIC was conducted as described elsewhere with modifications [56]. Briefly, 10 μg rPrP, 5 × 10^−6^ fold diluted RML-SBH, and *CpV* extract (0.001–0.1 mg/mL) were mixed in 100 μL of RT-QuIC buffer [0.5 M NaCl, 10 μM EDTA, 10 μM ThT, 0.002% SDS, 2% acetonitrile, 0.005% trifluoroacetic acid in 10 mM phosphate buffer (pH 7.4)]. The reaction mixture was incubated at 42 °C for 60 h with continuous shaking at 700 rpm in FLUOstar Omega Fluorescence reader (BMG Labtech, Ortenberg, Germany). Fibrils formation was measured in real-time every 15 min by detecting ThT fluorescence intensity.

### 4.4. PrP^Sc^ Stability Assay

RML-SBH that includes PrP^Sc^ was used to analyze the effect of *CpV* extract to PrP^Sc^ stability. The assay was performed as described in our previous publications with minor modifications [34]. Approximately 200 μg of RML-SBH was incubated with *CpV* extract (0.001–0.1 mg/mL) in PBS (pH7.4) with 1% NP-40 at 37 °C overnight with continuous agitation at 300 rpm. After digestion with 20 μg/mL PK (Roche, Basel, Switzerland), PK-resistant material in the reaction was analyzed using western blotting.

### 4.5. Cytotoxicity Assay

MTT assay was performed to determine the cytotoxicity of *CpV* extracts following the manufacturer’s instructions in the Cell Proliferation Kit I (MTT) (Roche, Switzerland). ScN2a cells, a murine neuroblastoma cell line chronically infected with mouse-adapted Chandler isolate of sheep scrapie prions [57], were grown in Dulbecco’s Modified Eagle’s Medium (Invitrogen, Waltham, MA, USA) with 10% fetal bovine serum (Corning, Corning, NY, USA), 1× Glutamax (Invitrogen, Waltham, MA, USA), 100 μg/mL penicillin and streptomycin (Invitrogen, Waltham, MA, USA) [58]. For experiments, cells were seeded 6 h before incubation with *CpV* extracts for 4 days without media change. The cells were immediately used for the assay.

### 4.6. Cell-Based PrP^Sc^ Assay

Anti-prion activity of *CpV* extracts was determined by measuring the level of PK-resistant PrP^Sc^ as described previously [58]. ScN2a cells were grown and incubated with *CpV* extracts in the same manner as described above. The cells were disrupted for lysate preparation using lysis buffer [20 mM Tris (pH 8.0), 150 mM NaCl, 0.5% Nonidet P-40 and 0.5% sodium deoxycholate] and the cell lysate was further analyzed using western blotting. After protein quantification with a Pierce BCA protein assay kit (ThermoFisher Scientific, Waltham, MA, USA), cell lysates (0.2 mg) were incubated with 20 μg/mL PK (Roche, Switzerland) at 37 °C for 1 h. The reaction was stopped using 2 mM phenylmethylsulfonyl fluoride (PMSF, Merck Millipore, Burlington, MA, USA), and the samples were centrifuged at 16,000× *g* at 4 °C for 1 h. The pellet was dissolved in a 4× sample loading buffer containing 8% sodium dodecyl sulfate (SDS, Sigma, St. Louis, MO, USA), 30% glycerol (Sigma, St. Louis, MO, USA), 0.02% bromophenol blue (Sigma, USA), and 8% β-mercaptoethanol (Sigma, St. Louis, MO, USA) in 0.25 M Tris (pH 6.8, Sigma, St. Louis, MO, USA). Then, the resulting mixture was boiled at 95 °C for 5 min and subjected to SDS-PAGE. The separated samples in the gel were transferred to Immobilon-P polyvinylidene difluoride membranes (Merck Millipore, Burlington, MA, USA), which were then blocked with 5% Difco skim milk (BD, Franklin Lakes, NJ, USA) in Tris-buffered saline with 0.1% Tween 20 (Sigma, St. Louis, MO, USA) at room temperature for 1 h. After overnight incubation with mouse monoclonal anti-PrP antibody 6D11 (diluted 1:30,000 fold, Biolegend, San Diego, CA, USA), the HRP-conjugated goat anti- mouse IgG (diluted 1:10,000 fold, Invitrogen, Waltham, MA, USA) antibody was used to incubate the membrane at room temperature for 1 h. The PK-resistant PrP^Sc^ bands were detected using Amersham ECL Prime Western Blotting Detection Reagent (Cytiva, Marlborough, MA, USA), and the signal was visualized using a Gbox Chemi XR5 image analysis system (Syngene, Cambridge, UK). Separately, the same amount (20 μg) of cell lysate without PK digestion was used for the immuno-detection of the β-actin and total PrP levels. The method was the same as that described above, while mouse monoclonal anti-β-actin antibody (Santa Cruz Biotechnology, Dallas, TX, USA) diluted to 1:5000 and mouse monoclonal anti-PrP antibody 6D11 (Biolegend, San Diego, CA, USA) diluted 1:30,000 were used as the primary antibodies, respectively. The signal intensity was quantified through densitometry using GeneTools software ver 1.5.0.0 (Syngene, Cambridge, UK).

### 4.7. Incubation Period Assay

To evaluate the effect of *CpV* extract on the disease’s course, the disease onset period was measured in a mouse model of prion disease. The animal experiments were performed under the approval of the Institutional Animal Care and Use Committee at Hanyang University (2020-0240). After a week of adaptation, 5-week-old female CD-1 mice (Samtacobio, Osan, Korea) were intraperitoneally inoculated with 50 μL of 1% (*w*/*v*) RML-SBH [33]. *CpV* extract (100–200 mg/kg) was administered from the day after prion inoculation 3 times a week for 4 weeks by gavage. Mice were kept under 12:12 light:dark room conditions until they showed obvious prion-related neuropathic signs, such as weight loss, starry coat, hunched posture, decreased movement and rigid tails. When a mouse exhibited two or more clinical signs, it was humanely euthanized using carbon dioxide, and the incubation period was counted for an individual mouse. The average incubation periods and standard error of the means of each group were used for comparison. Mice without such signs in the group that received *CpV* extract were observed until 452 days post-inoculation (dpi) and then euthanized. Prion-inoculated mice that received vehicle (10% DMSO in PBS) only served as control.

### 4.8. PrP^Sc^ Assay Using Brain and Spleen Tissue

The levels of accumulated PrP^Sc^ in the brain and spleen were measured using the method described previously [33]. The tissue homogenate (10%, *w*/*v*) in PBS was prepared using the silicon beads with OMNI Bead Ruptor 24 (PerkinElmer, Waltham, MA, USA). After brief centrifugation at 1000× *g* for 5 min at room temperature, the separated supernatant was quantified, and 0.2 mg of brain homogenate was mixed with 2% Sarkosyl PBS. PK digestion and western blotting were conducted using the same method described in the cell-based PrP^Sc^ assay section.

### 4.9. Detection of FDC Activation in the Spleen

Western blotting was performed to determine the level of follicular specific antigen FDC-M1 (also known as milk fat globule-EGF factor 8, MFGE-8). The procedures were the same as described in PrP detection without PK digestion. Spleen tissue homogenate was used for FDC-M1 (60 μg) and β-actin (30 μg) western blotting. For the detection of FDC-M1, rat monoclonal anti-mouse FDC-M1 (1:500 diluted, BD Pharmigen, San Diego, CA, USA) was used as the primary antibody. Biotinylated anti-rat IgG (1:500 diluted; Vector Laboratories, Burlingame, CA, USA) and streptavidin-HRP (1:10,000 diluted, Abcam, Cambridge, UK) were sequentially used. Detection of β-actin followed as described above.

### 4.10. Serum Chemistry

The samples were prepared from both prion-free, healthy and prion-infected, ill mice that received *CpV* extract or vehicle. The whole blood of mice was collected through cardiac puncture sampling before euthanasia, as describe elsewhere [59]. Serum was separated and tested directly using an automated clinical chemistry analyzer, Fuji DRI-CHEM 4000i (Fujifilm, Tokyo, Japan), and recommended colorimetric slides (Fujifilm, Japan). Long-term resistance to liver toxicity was confirmed by biochemical results for AST, ALT, ALP, and ALB.

### 4.11. Histology and Immunohistochemistry

The extracted mouse brain was fixed overnight in 4% paraformaldehyde (Sigma, St. Louis, MO, USA) in PBS at 4 °C, and the paraffin block was made by a tissue processor Citadel 2000 (ThermoFisher Scientific, Waltham, MA, USA). Paraffin-embedded tissues were cut into slices with a thickness of 6 μm using a microtome (hm340e, Thermo Scientific, Waltham, MA, USA). Hematoxylin and eosin (H&E) staining of the tissue sections was performed using Mayer’s hematoxylin and eosin-Y (Cancer Diagnostics Inc., Durham, NC, USA). Mounted brain tissue was observed with an optical microscope (Eclipse Ni-U, Nikon, Tokyo, Japan), and the histological images were obtained using an Axioscan Z1 slide scanner (Zeiss, Oberkochen, Germany). The degree of vacuolation was determined by vacuole number counts per unit area using Zen2 software (Zeiss, Oberkochen, Germany). Immunohistochemistry of PrP^Sc^, GFAP, and Iba-1 was performed using a M.O.M. immunodetection kit (Vector Laboratories, Burlingame, CA, USA) according to the manufacturer’s instructions. Briefly, the deparaffinized brain tissue was boiled in 10 mM sodium citrate buffer (pH 6.0) for 10 min and was then incubated in the quenching solution (0.3% H_2_O_2_) for 5 min. For the brain tissue to detect PrP^Sc^, incubation with 10 μg/mL PK was conducted in PBS for 10 min at 37 °C prior to the quenching step. After blocking with M.O.M. blocking reagent for 1 h, the mouse monoclonal anti-PrP antibody (6D11, diluted 1:3000-fold in M.O.M. diluent, Biolegend, San Diego, CA, USA) was applied for 2 days at 4 °C. For the brain tissue to detect GFAP and Iba-1, the mouse monoclonal anti-GFAP (diluted 1:500-fold in M.O.M. diluent, Sigma, St. Louis, MO, USA) and rabbit monoclonal anti-Iba-1 (diluted 1:1000-fold in M.O.M. diluent, Fujifilm Wako Chemicals, Japan) antibodies were used for the incubation for 1 h at room temperature. The common incubation step with the biotinylated anti-mouse IgG (diluted 1:200-250-fold, Vector Laboratories, Burlingame, CA, USA) was sequentially conducted for 1 h at room temperature. PBS was used for washing between each step. VECTASTAIN^®^ Elite ABC-HRP Kit, Peroxidase (Vector Laboratories, Burlingame, CA, USA) was used to detect biotinylated molecules. Detection of specific staining was performed using Vector DAB Substrate (Vector Laboratories, Burlingame, CA, USA). The number of GFAP- and Iba-1-positive nucleated cells per unit area were counted using Zen2 software (Zeiss, Oberkochen, Germany). The PrP^Sc^ intensity was determined using H-DAB in ImageJ software (NIH, Bethesda, MA, USA). The vacuolation, PrP^Sc^ accumulation, astrogliosis, and microglia activation were analyzed at three independent sites within the same area of the cortex, hippocampus, hypothalamus, striatum, and thalamus.

### 4.12. In Vitro Prion Infection Assay

To evaluate the effect of *CpV* extract on prion infection, the standard scrapie cell assay (SSCA) [60] was employed with minor modifications. N2a cells (2.4 × 10^4^ cells) were cultured in a 24-well dish in the presence of 0.9% (final concentration) RML-SBH for 4 days with or without 50 μg/mL *CpV* extract. N2a cells without prion infection were also cultured as a control. Then, the cells were maintained over several passages. At passages 4 and 10, 2.5 × 10^4^ cells were seeded and fixed on the activated opaque 96-well plate with 0.45 μm hydrophobic high protein binding Immobilon-P membrane (Merck Millipore, Burlington, MA, USA). The membrane underwent incubation sequentially with 5 μg/mL PK in RIPA lysis buffer (50 mM Tris, 150 mM NaCl, 1% sodium deoxycholate, 1% Nonidet P-40, and 0.1% SDS) for 90 min and 2 mM PMSF in PBS for 20 min. Then, it was exposed to 3 M guanidine thiocyanate for 10 min and thoroughly washed with PBS. After blocking with Superblock (ThermoFisher Scientific, Waltham, MA, USA) solution, PrP^Sc^ was specifically labeled with mouse monoclonal anti-PrP antibody 6D11 and alkaline phosphatase-conjugated mouse IgG (Abcam, Cambridge, UK) at the same concentrations used in western blot analysis. After color development using nitro-blue tetrazolium chloride/5-bromo-4-chloro-3’-indolyphosphate p-toluidine salt (NBT/BCIP) substrate (Sigma, St. Louis, MO, USA), the number of infected cells was analyzed using a S6 universal M2 ELISPOT reader ImmunoSpot (CTL, Shaker Heights, OH, USA) and ImmunoSpot software (CTL, Shaker Heights, OH, USA).

### 4.13. Statistical Analysis

The length of the incubation period of each animal group was described using the Kaplan–Meier survival analysis, and the significance of the differences among groups that received various concentrations of *CpV* extract were evaluated with two-side log-rank tests using SPSS27 software (IBM, Armonk, NY, USA). The statistical significance of difference in other experimental data was analyzed using Student’s *t*-test.

## 5. Conclusions

Together with the results of a series of in vitro and in vivo experiments, we concluded that *CpV* extract effectively mitigated prion propagation and modified the course of prion diseases by decreasing the level of PrP^Sc^ and preventing neuropathological deteriorations. *CpV* extract is interesting to be further explored and validated toward the development of an agent that modulates prion propagation.

## Figures and Tables

**Table 1:** The average incubation periods and the disease onset frequency of prion-infected mouse groups that received *CpV* extract ^1^.

Group	Incubation Period Mean ± SEM (Days)	n/n_0_	*p* Value (versus Vehicle)
vehicle	202.4 ± 4.44	9/9	
*CpV* Ext 100 mg/kg	209.0 ± 6.3	9/9	n.s.
*CpV* Ext 200 mg/kg	212.8 ± 4.71 ^†^	5/8	0.009 (n.s. ^†^)

^1^ *CpV* extract (100 and 200 mg/kg) was administered intraperitoneally twice a week for four weeks staring from dpi 1 to wild-type CD-1 mice inoculated with RML prions. SEM, standard error of the mean; n, number of mice diagnosed ill with prion disease; n_0_, number of mice inoculated with prions; †, The mean incubation period ± SEM and *p* value were calculated from the dataset of those five prion-ill mice in the group; n.s., statistically not significant.

## Data Availability

The datasets used and/or analyzed during the current study are available from the corresponding author on reasonable request. A part of the data generated or analyzed during this study is included in this published article and its Supplementary Information Files.

## Supplementary Materials

The supporting information can be downloaded at: https://www.mdpi.com/article/10.3390/ijms24010182/s1.
