# Supplementary material for: The Effect of Curcuma phaeocaulis Valeton (Zingiberaceae) Extract on Prion Propagation in Cell-Based and Animal Models
Source: Int J Mol Sci. 2022 Dec 22;24(1):182. doi: 10.3390/ijms24010182 (PMC9820341; doi:10.3390/ijms24010182)
Supplement: Supplementary file 1 [file ijms-24-00182-s001.zip › ijms-1961336-supplementary.pdf]

Supporting Information

Curcuma phaeocaulis Valeton (Zingiberaceae) extract suppresses prion disease in cell-based and animal models

Sungeun Lee, Hakmin Lee, Jaehyeon Kim, Jihoon Kim, Eun Mei Gao, Yoonjeong Lee, Miryeong Yoo, Trang H. T. Trinh, Jieun Kim, Chul Young Kim[†] and Chongsuk Ryou[†]

Department of Pharmacy, College of Pharmacy, and Institute of Pharmaceutical Science & Technology, Hanyang University

55 Hanyangdaehak-ro, Ansan, Gyeonggi-do, 15588, Republic of Korea,

Running title: Anti-prion efficacy of *CpV* extract

[†] Co-corresponding authors: Chongsuk Ryou, Ph.D. and Chul-Young Kim, Ph.D.

Address: 55 Hanyangdaehak-ro, Sangnok-gu, Ansan-si, Gyeonggi-do, 15588, Republic of Korea.

Tel: (+82) 31-400-5811

Fax: (+82) 31-400-5958

E-mail: cryou2@hanyang.ac.kr (C.R.), chlykim@hanyang.ac.kr (C. Y. K.)

Supplementary Figures

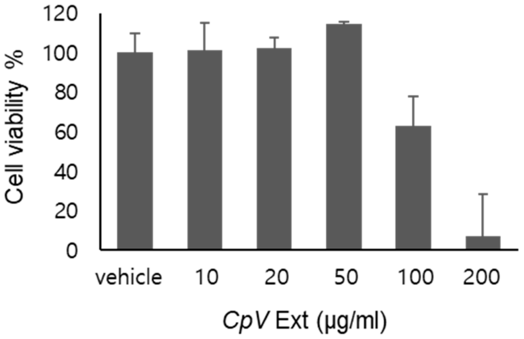

Figure S1 Cytotoxicity of *CpV* extract in ScN2a cells. Cytotoxicity of *CpV* extract was measured in ScN2a cells by MTT assay. The cells were incubated with varying concentrations (0 – 200 μg/ml) of *CpV* extract for 4 days. The assay was performed in triplicate. Cell viability was not affected up to 50 μg/ml of *CpV* extract.

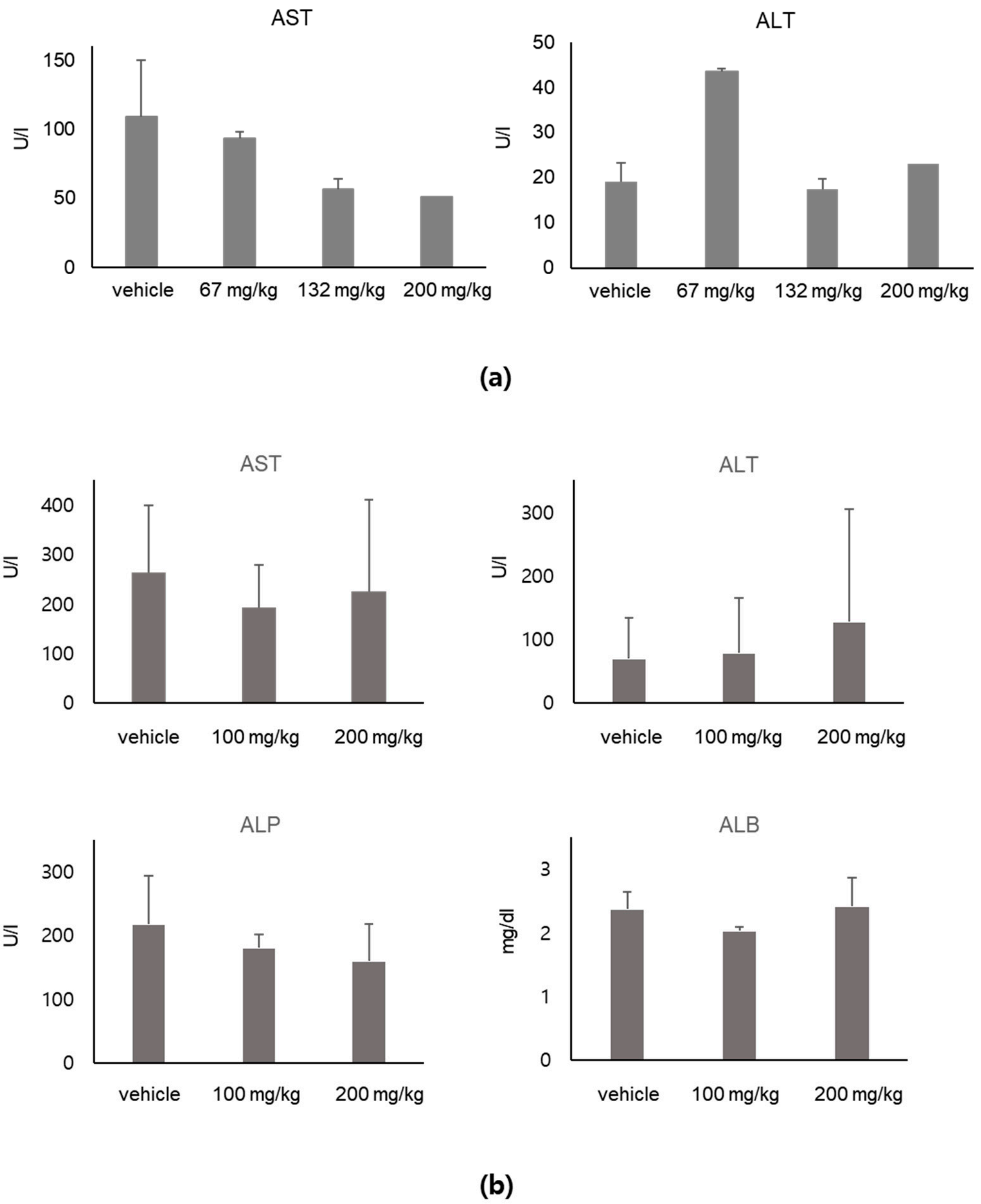

Figure S2 Toxicity of *CpV* extract in mice. (a) Serum biochemistry of healthy, uninfected mice that received *CpV* extract. Serum was collected from mice at the day after a month-administration of *CpV* extract was completed. (b) Serum biochemistry of prion-infected mice that received *CpV* extract. The samples were prepared from mice that displayed the clinical signs of prion disease at euthanasia.

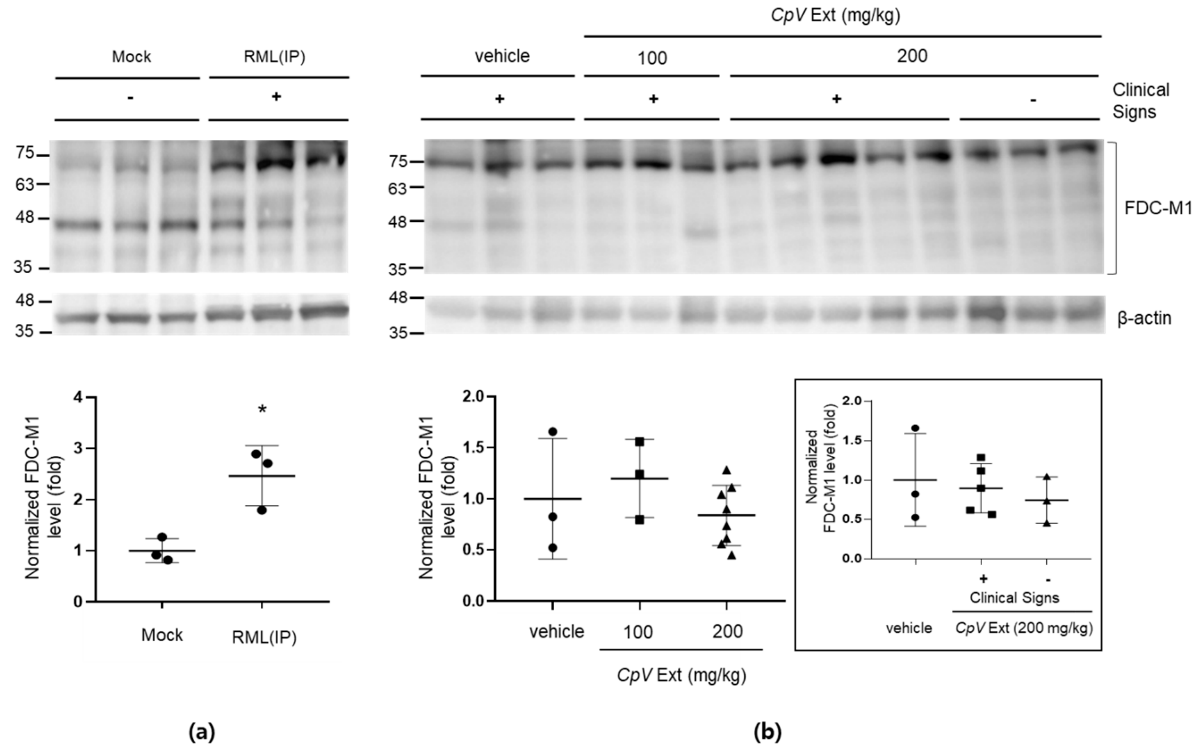

Figure S3 The effect of *CpV* extract to FDC-M1 in mouse spleen. (a) The level of FDC-M1 in the spleen of mice intraperitoneally infected with mock inoculum and RML prions. *, p=0.016. (b) The level of FDC-M1 in the spleen of mice that were intraperitoneally infected with RML prions RML prion-infected and administered with *CpV* extract and vehicle. The spleen samples were collected at the end point of individual mouse. β-actin was used a loading control. The FDC-M1 level normalized by the β-actin level was plotted. The subgroups with (+) or without (-) clinical signs within 200 mg/kg group were divided and compared individually to control group (inset).

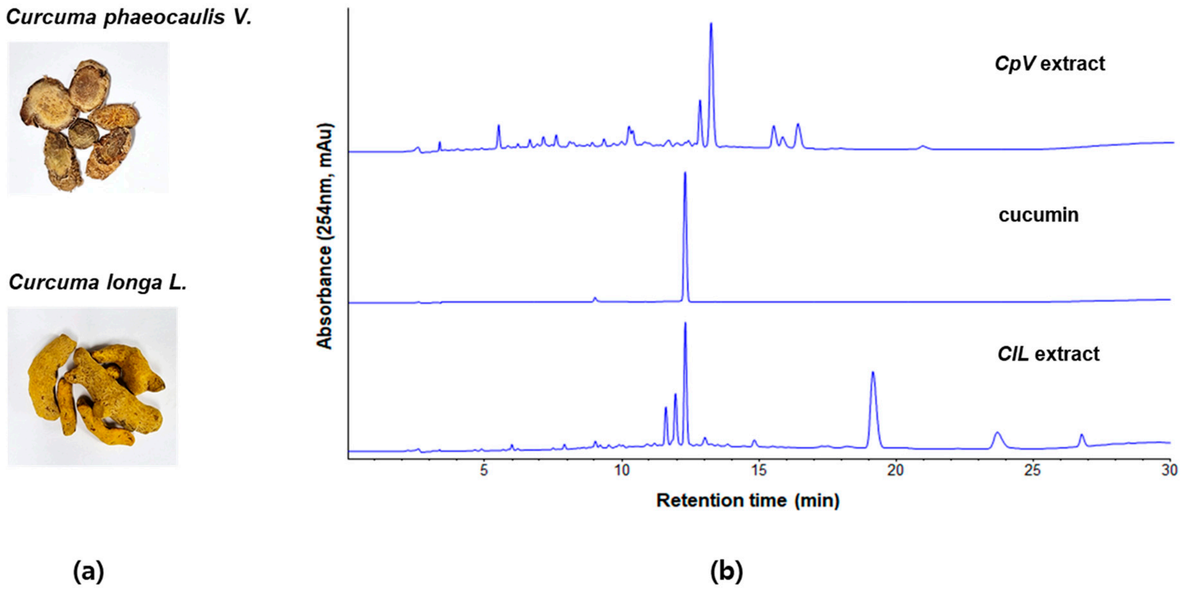

Figure S4 HPLC chromatogram of *CpV* extract used in this study. (a) Rhizomes of *Curcuma phaeocaulis Val*. (*CpV*) and *Curcuma longa L*. (*ClL*). (b) HPLC chromatogram of *CpV* and *ClL* extracts and purified curcumin (Sigma-Aldrich, Korea). Curcumin, a well-known diarylheptanoid in curry, is not contained in *CpV* extract, but abundantly included in *ClL* extract as one of the major components.
